# Droplet-Interlaced Generator with On-Chip Metal–Liquid Micromirrors for Enhanced Microfluidic Absorbance Detection

**DOI:** 10.3390/bios16040202

**Published:** 2026-04-02

**Authors:** Haobo Liu, Laidi Jin, Zehang Gao, Chuanjin Cui, Yongjie Yu, Fei Deng, Xiuli Gao, Jianlong Zhao, Shengtai Bian, Shilun Feng

**Affiliations:** 1 School of Electrical Engineering, North China University of Science and Technology, Tangshan 063000, China; 15392541653@163.com (H.L.); chuanjincui@126.com (C.C.); 2 State Key Laboratory of Transducer Technology, Shanghai Institute of Microsystem and Information Technology, Chinese Academy of Sciences, Shanghai 200050, China; gaozh@shanghaitech.edu.cn (Z.G.); gxl@mail.sim.ac.cn (X.G.); jlzhao@mail.sim.ac.cn (J.Z.); 3 Department of Stomatology, Shanghai Municipal Hospital of Traditional Chinese Medicine, Shanghai University of Traditional Chinese Medicine, Shanghai 200071, China; jld100302@163.com; 4 School of Materials Engineering, Changzhou Vocational Institute of Industry Technology, Changzhou 213164, China; yu.yongjie@hotmail.com; 5 Graduate School of Biomedical Engineering, Faculty of Engineering, University of New South Wales, Sydney, NSW 2052, Australia; fei.deng@unsw.edu.au; 6 Shanghai Frontier Innovation Research Institute, Shanghai 201108, China; 7 Xiangfu Laboratory, Jiaxing 314100, China; 8 Agriculture and Forestry Artificial Intelligence Research Institute, Fujian Agriculture and Forestry University, Fuzhou 350002, China; 9 College of Mechanical and Electrical Engineering, Fujian Agriculture and Forestry University, Fuzhou 350002, China

**Keywords:** peristaltic pump, microfluidics, optical fiber, absorbance detection

## Abstract

Droplet microfluidics has been widely used in biological, chemical, and medical research owing to its advantages of miniaturization, high throughput, and low reagent consumption. However, limited sensitivity and optical path length in on-chip absorbance detection remain major challenges for droplet-based microfluidic analysis. Traditional absorbance detection suffers from low sensitivity due to the extremely short optical path in microfluidic channels, while existing optical path extension methods have drawbacks such as complex fabrication, easy droplet rupture, or strict incident angle requirements. To address these issues, this study developed a droplet microfluidic absorbance detection platform integrating optical fibers, on-chip micromirrors, external fluidic actuation, and an absorbance detection module. Microchannel sidewalls filled with low-melting-point metal act as mirrors; the multi-reflection optical path, combined with optical fibers and micromirrors, compensates for insufficient light manipulation and effectively extends the absorption path length, improving sensitivity and accuracy. Using this method, the detection limit for methylene blue solution was 20 μM, and the sensitivity for *Escherichia coli* (*E. coli*) suspension was doubled compared with traditional Nanodrop OD600 measurement. This device features low fabrication difficulty and cost and stable detection, providing a proof-of-concept strategy for enhanced absorbance detection in droplet microfluidic systems.

## 1. Introduction

In recent years, droplet microfluidics has attracted widespread attention in various research fields due to its distinctive advantages. This technology utilizes immiscible two-phase fluids to fabricate emulsified droplets [1], and reagents are encapsulated into individual droplets for reaction and detection [2], thereby achieving miniaturization and high-throughput operation [3] of the system. Each droplet can serve as an independent reaction vessel, effectively preventing cross-contamination between samples, reducing the consumption of reagents and samples [4,5], and remarkably improving reaction reliability and detection sensitivity. Over the past decade or so, droplet-based microfluidics has been successfully applied in multiple fields, providing an efficient, flexible, and highly controllable platform for biological [6], chemical [7], and medical research [8].

Despite significant advancements in the miniaturization of fluid manipulation, the development of on-chip detection and analysis technologies has lagged relatively behind [9], emerging as a critical bottleneck restricting the practical applications of droplet microfluidics. Conventional detection devices often rely on off-chip optical systems (such as fluorescence microscopes [10] and laser-induced fluorescence detectors [11]) for signal readout. These devices are bulky and costly, making it difficult to achieve miniaturized integration with microfluidic chips and limiting their deployment outside well-controlled laboratory environments which raises the threshold for researchers to apply microfluidic technologies.

To address the challenge of integrating detection systems, researchers have begun exploring the direct integration of optical components onto microfluidic chips to construct on-chip optical detection configurations. Achieving on-chip light guiding is a core link in building such systems, which is currently mainly realized through two approaches: waveguides [12] and optical fibers [13]. As a common on-chip light guiding component, waveguides exhibit excellent structural integration performance, enabling high compatibility with microfluidic chips. They can also be flexibly designed to allow arbitrary light propagation paths within the chip according to detection requirements, offering strong light manipulation capabilities. However, their fabrication process requires precise control of material refractive index differences, resulting in complex craftsmanship and high costs. Meanwhile, waveguides are susceptible to factors such as chip surface defects and temperature variations [14], leading to unstable light guiding efficiency. These factors have to a certain extent limited the promotion of waveguides in cost-sensitive and large-scale laboratory applications. Compared with waveguides, although optical fibers have weaker light manipulation capabilities in microfluidic on-chip detection, they possess significant advantages: mature manufacturing processes, low production costs, and high cost-effectiveness [15]. Additionally, optical fibers can be directly inserted into microfluidic channels to establish optical paths, featuring simple and rapid operation without the need for complex microfabrication processes, thus facilitating rapid laboratory-level integration with microfluidic chips. Thanks to these cost-effective and convenient integration characteristics, optical fibers have been combined with various optical detection methods such as absorbance detection [16], fluorescence detection [17], and Raman spectroscopy detection [18] for application in microfluidic on-chip detection. Among these methods, absorbance detection has been widely used in fields such as chemical analysis and biomolecular quantitative detection due to its prominent advantages, including simple operation, no need for sample labeling [19], and low detection costs. However, traditional absorbance detection also faces challenges in microfluidic chips: the optical path length L of conventional cuvettes is usually fixed (e.g., 10 mm), but the depth and width of microfluidic chip channels are relatively shallow [20]. When light passes vertically through the channels, L becomes extremely short, leading to a significant reduction in detection performance.

To address the sensitivity issue caused by insufficient optical path length, researchers have continuously attempted to improve the absorbance detection sensitivity of microfluidic chips by extending the optical path over the past few decades [21], including the use of collimation systems [22] and multi-reflection systems [23]. Tianjin Yang et al. [24] proposed a Z-shaped detection path design integrated with a PDMS liquid-core waveguide. By ingeniously designing the microchannel into a Z-shaped structure, the interaction length between light and the sample was further increased, thereby effectively extending the optical path. This method expanded the effective optical path to 700–800 μm (enhanced by more than one order of magnitude) and optimized the detection limit of fluorescein to 400 nM. Meanwhile, it realized the simultaneous collection of absorbance and fluorescence signals, providing a new technical approach for high-sensitivity detection. However, when droplets pass through such microchannels with curved structures (including Z-shaped and U-shaped), they are prone to shear forces generated at the channel corners, leading to droplet rupture and thus affecting the stability and accuracy of detection. Therefore, Llobera et al. [25] proposed constructing a multi-internal reflection (MIR) system containing air mirrors based on polydimethylsiloxane (PDMS). Utilizing the refractive index differences between PDMS, air, and buffer solution, light satisfies the total internal reflection condition at the air mirrors to extend the optical path. Among them, the RMIR configuration reduced the limit of detection (LOD) of fluorescein to 41 nM by adding a third air mirror and effectively controlled the flow cell volume. Nevertheless, this system also has certain drawbacks: some microchannels form “dead volumes” where no light propagates, increasing the volume without improving sensitivity, and multiple reflections of the light beam are prone to divergence, limiting detection performance. To solve the dead volume and beam divergence problems of the air mirror system, Yanagisawa et al. [26] proposed introducing aluminum reflective mirrors on the upper and lower surfaces of the microchannel. The effective optical path was extended through multiple reflections of light between the aluminum mirrors. In experiments, when the axial overlap length of the mirrors L = 4 mm and the chip tilt angle φ = 7°, the detection sensitivity was 52 times higher than that of the mirror-free structure, and it was compatible with commercial microplate readers. However, this scheme also has certain limitations: it has strict requirements on the incident angle of light. The light beam must be incident at a specific angle to ensure stable multiple reflections between the upper and lower aluminum mirrors and final emission from the exit aperture. Therefore, developing micromirror-based optical path extension strategies with low fabrication complexity and relaxed incident-angle constraints remains an important technical challenge.

**Figure 1:**
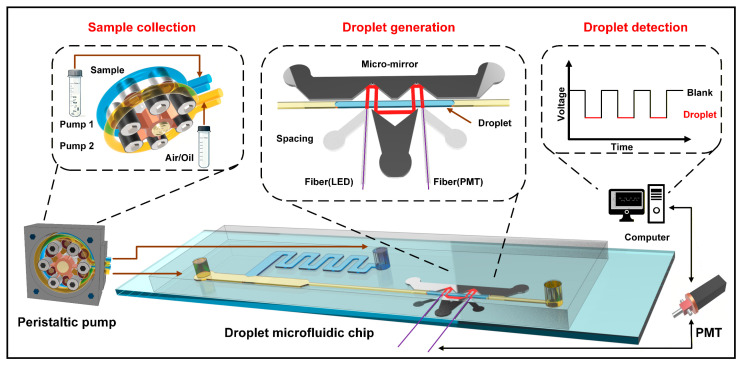
Droplet Microfluidics-Based Sampling and Detection Device.

In this work, we developed a lab-based droplet microfluidic absorbance detection platform combining optical fibers, micromirrors, external fluidic actuation, and absorbance detection. The system workflow is illustrated in Figure 1: the continuous phase (air) and dispersed phase (sample solution) are alternately pumped into the microfluidic chip by external fluidic actuation. At the junction of the T-shaped microchannel, the sample solution is sheared into droplets by air and then enters the strip-shaped detection zone. Pre-fabricated on-chip micromirrors (fabricated via low-melting-point metal filling) are placed at both ends of the detection zone. The incident light (from the excitation optical fiber) undergoes vertical reflection by the microfabricated mirrors, passes through the detection channel multiple times, and is finally collected by the emission optical fiber. The absorbance of the solution is detected by measuring the change in light intensity output from the emission optical fiber. Herein, a photomultiplier tube (PMT) accurately detects this light intensity change, converts it into a corresponding electrical signal, and transmits the signal to a LabVIEW-based upper computer. Through the analysis and processing of the electrical signal, the absorbance of the target substance in the droplets can be calculated according to the Beer-Lambert law, thereby realizing the quantitative analysis of the concentration of specific components in the droplets. In addition, we systematically characterized the performance of the peristaltic pump and the microfluidic chip. The optimal parameters for droplet generation were determined through experimental optimization, and the best design scheme for the on-chip micromirrors was clarified, providing key support for improving the overall performance of the device. Furthermore, the combination of optical fibers with such micromirrors not only compensates for the shortcomings of optical fibers in microfluidic on-chip detection (e.g., weak light manipulation capability and inability to achieve complex light regulation through flexible optical path design like waveguides) but also extends the propagation path of the light beam in the droplets and increases the effective detection optical path via multiple reflections of the light beam by the micromirrors, ultimately enhancing the sensitivity of absorbance detection in a controlled laboratory setting.

## 2. Materials and Methods

### 2.1. Optical Fiber Integration and Optical Configuration

The coating (3 mm) and cladding of all optical fibers were stripped off using a three-port Miller clamp (Guangdong Shixiantong Technology Co., Ltd., Shenzhen, Guangdong, China, leaving only the core. The stripped core was cleaved with a fiber cleaver (HB-08, Guangdong Shixiantong Technology Co., Ltd.) to obtain a flat surface at the tip, and the flatness of the core end was confirmed via microscopic imaging (Olympus, Tokyo, Japan). The optical fibers were manually inserted into the detection zone of the chip through fiber guiding structures and fixed on the glass slide (Jiangsu Shitai Laboratory Equipment Co., Ltd., Nanjing, China) with instant adhesive. The excitation fiber (FC fiber patch cord, multimode, Dongguan Xinruiguang Technology Co., Ltd, Dongguan, China) has a cladding diameter of 220 μm, a core diameter of 200 μm, and a numerical aperture (NA) of 0.22. It is connected to an LED fiber-coupled light source (488 nm and 600 nm, Shaanxi GroWom Optoelectronics Technology Co., Ltd., Xi’an, China) with an FC interface. The detection fiber, with the same parameters as above, is connected to the FC interface of a photomultiplier tube (H10722-110, Hamamatsu Photonics Trading (China) Co., Ltd., Shanghai, China), which converts the optical signal into an electrical signal and transmits it to the upper computer (LABVIEW) for subsequent analysis.

### 2.2. Chip Fabrication

#### 2.2.1. Microfluidic Chips

**Figure 2:**
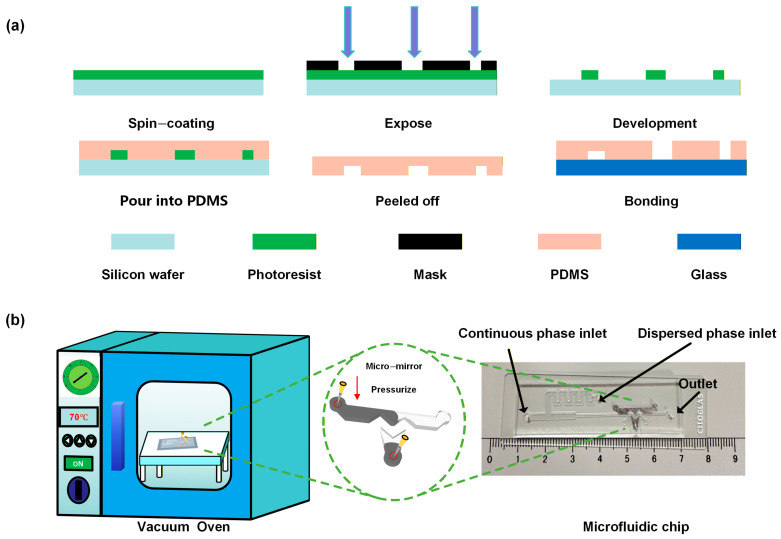
(**a**) Fabrication Process Flowchart of the Microfluidic Chip. (**b**) Fabrication Process Flowchart of On−chip Micro-reflectors.

The droplet generation chips are fabricated using a standard soft lithography process. Firstly, the microchannels are patterned using CleWin software 5.0. Next, SU-8 3050 negative photoresist (Microchem, Inc. (Newton, MA, USA)) is spin coated onto a clean silicon wafer (Mesk Electronic Materials Co., Ltd., Suzhou, China) (1000 rpm for 30 s), resulting in an 100 µm thick photoresist layer. The wafer with the photoresist coating was pre-baked on a hotplate (Camet Functional Ceramics, Inc., Atlanta, GA, USA) at 95 °C for 30 min. This spin-coating and pre-baking process was repeated once more, resulting in a final photoresist layer thickness of 300 μm. MicroWriter ML3 maskless lithograph (Durham Magneto Optics, Durham, UK) to form the microchannels for droplet generation. Subsequently, the wafer was baked at 95 °C for 20 min, followed by removal of the unexposed photoresist using a developer solution (Luoyang Meyer Trading Co., Ltd., Luoyang, China). For device fabrication, the PDMS base and curing agent (Dow Corning Corporation, Midland, MI, USA) were mixed at a weight ratio of 10:1. After degassing, the mixture was poured onto the SU-8 photoresist-coated silicon master mold. It was then heated on a hotplate at 90 °C for 2 h. The cured PDMS structure was peeled off from the silicon wafer, and 1 mm-diameter access holes were punched at designated positions using a biopsy punch (Integra Miltex, Inc., York, PA, USA). Thereafter, both the PDMS chip and glass slide were treated in a plasma cleaner (Chengdu Mingheng Technology Development Co., Ltd., Chengdu, China). for approximately 1 min, then bonded together and baked in an oven (Shanghai Yiheng Scientific Instruments Co., Ltd., Shanghai, China) at 90 °C for 4 h to complete chip fabrication (Figure 2a). All microchannels have a height of 300 μm, with a main channel width of 1000 μm and a detection zone width of 500 μm.

#### 2.2.2. On-Chip Micromirrors

The microchannels of the microfluidic chip were designed, and the droplet optical detection zone was planned—including the required positions of the incident and exit optical fibers, as well as the shape and positions of the micromirrors. Channels for the micromirrors were arranged in areas that do not interfere with the optical path. A 47 °C low-melting-point alloy liquid metal thermal conductive silicone grease (Yantai Oulang Automation Technology Co., Ltd., Yantai, China) was used as the material for fabricating the on-chip micromirrors. First, the metal was heated in an oven to melt it into a liquid state. Subsequently, a 10 μL pipette tip was used to aspirate the liquid metal, which was then cooled to room temperature for solidification. Next, the pipette tip loaded with the solidified metal was inserted into the inlet of the micromirror zone on the microfluidic chip, and the chip with the pipette tip was placed in a vacuum drying oven (Shanghai Jinghong Instrument Co., Ltd., Shanghai, China) preheated to 70 °C. The oven was activated for heating while vacuum was applied. After maintaining this condition for several minutes, the vacuum was released and the chip was removed. During this process, the re-melted liquid metal flowed along the pre-designed microchannels and completely filled the designated area of the micromirrors. Finally, the chip was cooled to room temperature to induce solidification of the liquid metal, thereby completing the fabrication of the micromirrors (Figure 2b).

### 2.3. Microfluidic Actuation

An assembled peristaltic pump was used as the liquid driving device, consisting of a 42-step motor (0.7 N, Wenzhou Yuhui Electric Motor Co., Ltd., Wenzhou, China), a driver (TB6600, Wenzhou Pufei Trade Co., Ltd., Wenzhou, China), two complementary peristaltic pump heads (PC, 6 rollers, Huizhou United Zhongwei Technology Co., Ltd., Huizhou, China), and pump tubes (silicone, inner diameter of 0.4 mm, Huizhou United Zhongwei Technology Co., Ltd.). The two pump heads were independently used for delivering the continuous phase and dispersed phase liquids, and connected to the inlets of the microfluidic chip via stainless steel needles. By leveraging the discrete pulsation of conventional peristaltic pumps and upgrading the functions of the two pump heads to deliver two types of liquids separately, this alternating pumping mode enables the generation of more uniform droplets. Additionally, the roller extrusion design reduces wear and tear, extends the service life of the hoses, and lowers maintenance costs. Notably, the total cost of the entire actuation system is approximately 600 RMB, which significantly reduces the overall cost (see Table S1).

### 2.4. Reagents and Microbial Culture

Air was used as the continuous phase in all experiments. For chip performance testing, methylene blue solution (1%, Yida Technology (Quanzhou) Co., Ltd., Quanzhou, China) at specified concentrations in deionized water was employed as the dispersed phase. In microfluidic demonstration experiments, the *Escherichia coli* (*E. coli*) O157:H7 (ATCC, 43888) bacterial suspension diluted with PBS (1×, pH 7.2–7.4, Shanghai Titan Technology Co., Ltd., Shanghai, China) was used as the dispersed phase.

In microbial culture, first, thoroughly wipe the surface of the ultra-clean workbench (Shanghai Shangpu Instrument Equipment Co., Ltd., Shanghai, China) and all items to be placed inside with alcohol-soaked cotton. Turn on the ultraviolet (UV) lamp for sterilization for 30 min, then turn off the UV lamp and switch on the fan and lighting. Heat the inoculating loop (Shanghai Titan Technology Co., Ltd., Shanghai, China) to red-hot over the flame of an alcohol lamp; after cooling, gently pick a single colony from the tryptic soy agar (TSA) medium (Nantong Kaiheng Biotechnology Development Co., Ltd., Nantong, China) (avoiding edge or abnormally shaped colonies). Inoculate the colony into 9 mL of tryptic soy broth (TSB) (Nantong Kaiheng Biotechnology Development Co., Ltd., Nantong, China) in a sterile test tube, and shake gently to disperse the bacterial cells. Place the inoculated test tube in a constant-temperature incubator (Shanghai Rundu Biotechnology Co., Ltd., Shanghai, China) at 37 °C, and culture with shaking at 150 rpm for 12–18 h until the bacterial suspension becomes turbid. The obtained bacterial suspension is diluted with PBS buffer to different concentrations for subsequent tests within the laboratory setting.

## 3. Results and Discussion

### 3.1. Performance Analysis of the Peristaltic Pump

As a classic fluid delivery device, the core principle of a peristaltic pump lies in the periodic compression and release of an elastic pump tube, thereby driving the directional flow of fluid inside the tube. In a conventional single-pump-head configuration, a motor drives a series of annularly distributed rollers on the pump head to rotate sequentially. When the rollers rotate, they crush and occlude the underlying pump tube segment, forming a temporarily sealed “fluid chamber.” With the continuous movement of the rollers, this compressed fluid chamber moves forward along the pump tube. After the rollers pass, the pump tube reverts to its original shape due to elasticity, generating local negative pressure that draws subsequent fluid from the inlet end. This process repeats cyclically to achieve continuous fluid delivery. However, during the gap between successive rollers, the flow rate experiences a brief decrease as the pump tube recovers its shape to aspirate fluid, resulting in a non-steady linear output flow rate of the fluid. Therefore, this working mode based on discrete roller compression inevitably leads to periodic pulsation of the output flow rate, which in turn affects the stability of experiments in laboratory microfluidic setups.

To address the flow rate pulsation issue of conventional peristaltic pumps with a single pump head, traditional peristaltic pump technology has been specifically improved by adopting a “pair of interlaced peristaltic pump heads” design, where both pump heads are used to deliver the same type of fluid. The core principle lies in the interlaced movement of the pump heads, enabling their tube-compressing actions to complement each other: when the rollers of one pump head are in the phase of compressing the pump tube and pushing the fluid, the rollers of the other pump head are exactly in the phase of leaving the pump tube and allowing the tube to recover its shape for fluid aspiration. Through the precise coordination of this action timing, the flow rate pulses output by the two pump heads can offset each other, thereby significantly reducing the fluctuation amplitude of the overall output fluid flow rate and making it approach a stable linear flow rate.

**Figure 3:**
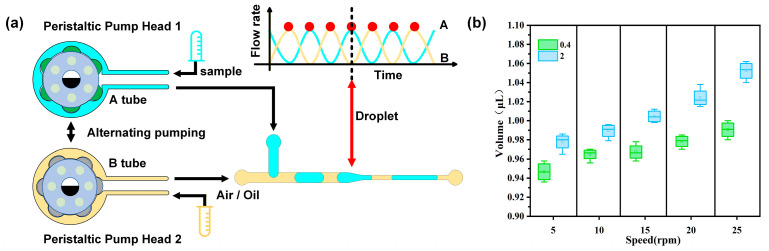
(**a**) Schematic Diagram of the Working Principle of the Peristaltic Pump Head. (**b**) Characterization Diagram of Droplets Generated under Different Motor Speeds and Pump Tube Inner Diameters.

Unlike traditional dual-pump-head peristaltic pumps, our peristaltic pump, while also adopting a “pair of interlaced peristaltic pump heads” structure, differs in design philosophy. Instead of aiming to simply smoothing flow for single-fluid delivery, it actively utilizes such discrete pulsations and upgrades the functions of the two pump heads to deliver two types of fluids separately, as shown in Figure 3a. Each pump head is equipped with six rollers. The two pump heads are mounted in a mechanically interlaced manner and their phases are controlled by the same drive system. The corresponding Tube A (for delivering the continuous phase) and Tube B (for delivering the dispersed phase) generate a precisely coordinated delivery rhythm with the interlaced pulsations of the pump heads: when the flow rate of Tube A reaches a peak, that of Tube B drops to a trough; when Tube A reaches a trough, Tube B instantly surges to a peak, forming a pulsed delivery. At the peak of each curve, there is a corresponding red dot, representing the moment when the dispersed phase is pulse-injected into the continuous phase. In this way, the discrete pulsations of the dual pump heads are not offset but instead serve as precisely timed delivery cues, enabling the two fluids to precisely cooperate in the T-shaped channel. When the dispersed phase fluid enters the junction region, under the combined effects of the shear force from the continuous phase fluid and interfacial tension, the interface of the dispersed phase undergoes stretching, necking, and final breakup to form stable droplets.

The peristaltic pump was connected to the microfluidic chip, with air as the continuous phase and pure water as the dispersed phase. Droplet generation was performed under different motor rotational speeds (5–25 rpm) and with peristaltic pump tubes of different inner diameters (0.4 mm and 2 mm). Each experimental condition was independently repeated three times, and 30 droplets were randomly selected in each experiment for measurement and averaging. The droplets were then imaged and characterized using a fluorescence inverted microscope (Figure 3b). The test results showed that the size of the generated droplets varied with different rotational speeds and pump tube inner diameters: higher rotational speeds and larger tube inner diameters led to larger droplets. The droplet volume ranged from 0.95 to 1.05 μL, enabling the generation of droplets of desired sizes according to experimental requirements. In subsequent experiments, to ensure that the incident light, after vertical reflection by the microfabricated mirrors, could completely pass through the interior of the droplets and be collected by the exit optical fiber when the droplets flowed through the on-chip detection zone channel, the motor rotational speed was set to 10 rpm and the pump tube inner diameter was set to 0.4 mm.

### 3.2. Verification of Chip Absorbance Performance

Notably, in theory, the incident light is a perfectly parallel beam without divergence, and the only reason for the reduction in light intensity is absorption by the light-absorbing substances in the sample. Therefore, according to the Beer-Lambert law, under the condition that the molar absorption coefficient and sample concentration remain constant, the absorbance is proportional to the optical path length—the longer the optical path length, the higher the absorbance. Thus, increasing the optical path length can significantly improve detection sensitivity, facilitating the measurement of low-concentration samples. The absorbance A is given by the following equation:

(1) $$A=\varepsilon \times c\times l=-\log_{10}\left(I_{0}/I\right)$$

where ε is the molar absorption coefficient, c is the sample concentration, I is the optical path length, $I_{0}$ is the transmitted light intensity, and I is the incident light intensity.

In this experiment, the output voltage of the PMT exhibits a linear relationship with the light intensity I (I = KV), where K is a constant coefficient). The voltage $V_{0}$ under the blank state (no droplets) corresponds to the transmitted light intensity $I_{0}$ ($I_{0}$ = K$V_{0}$). According to the original definition of absorbance, substituting the above linear relationship leads to the cancellation of the coefficient K, and finally the absorbance-voltage calculation formula applicable to the experimental system is derived:

(2) $$A=-\log_{10}\left(V_{0}/V\right)$$

where $V_{0}$ is the blank voltage and V is the real-time voltage.

However, in practical processes, when the optical path length increases to a certain extent, non-absorptive losses caused by divergence, scattering, and interface reflection increase sharply. This results in an extremely weak optical signal reaching the detector, which contains a large number of non-sample-related contributions and deviates significantly from the true absorbance generated solely by sample absorption, leading to inaccurate measurement results. Therefore, in practical applications, it is necessary to balance the optical path length to ensure sensitivity while avoiding excessive optical losses.

In this experiment, four microfluidic chips with different structures were designed and fabricated. Their core feature is that the incident light undergoes 0 to 3 reflections inside the chip by changing the shape of the mirror channels, thereby achieving four different effective optical path lengths: 0.5 mm, 1 mm, 2 mm, and 3 mm. By analyzing and processing the voltage signals collected by the respective exit optical fibers, the optimized chip configuration was determined.

To verify the chip performance, methylene blue solutions with concentrations ranging from 0.02 mM to 3.2 mM were encapsulated into droplets through the alternating pumping of the peristaltic pump, and experiments were conducted using microfluidic chips with optical path lengths of 0.5 mm, 1 mm, 2 mm, and 3 mm respectively. Pure water was used as the blank control group in the experiments. When using microfluidic chips with different optical path lengths, the non-absorptive loss of light intensity increases significantly as the number of reflections of the incident light gradually increases, leading to a subsequent decrease in the intensity of the emitted light signal. To ensure that the attenuated signal can be effectively detected, the sensitivity (gain) of the PMT connected to the exit optical fiber needs to be correspondingly increased with the increase in the number of reflections to compensate for the signal loss, thereby ensuring the reliability and consistency of the detection signals under different optical path conditions.

**Figure 4:**
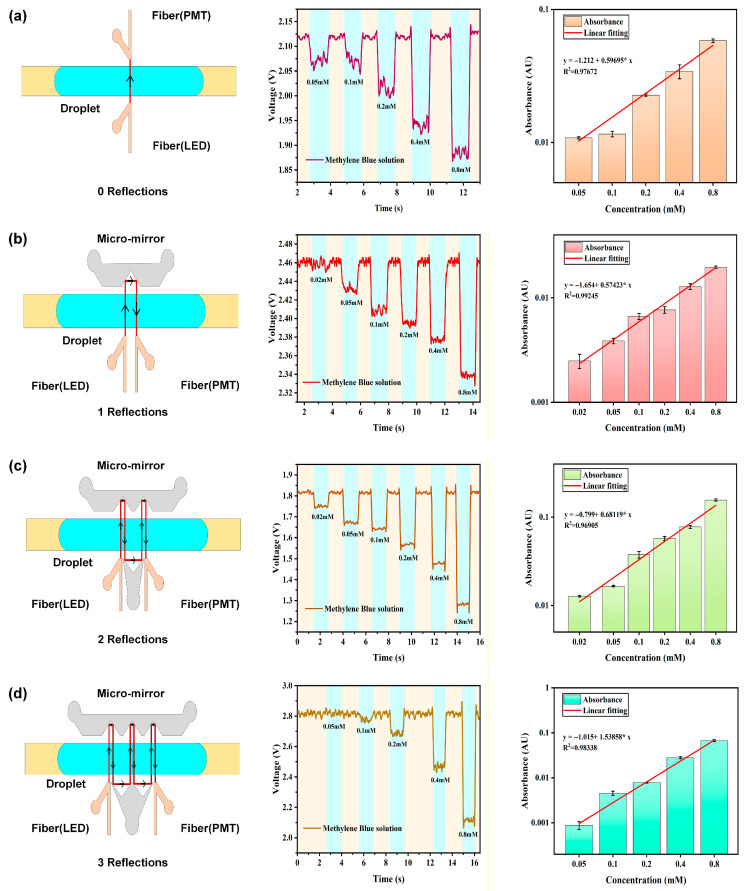
(**a**) The left panel shows the voltage fluctuation curves generated when droplets containing solutions of different concentrations pass through the detection zone of the chip without reflection (0 reflections). The middle panel presents the linear regression analysis of the data. The right panel illustrates the schematic diagram of no reflection (0 reflections). (**b**) The left panel shows the voltage fluctuation curves generated when droplets containing solutions of different concentrations pass through the detection zone of the chip without reflection (1 reflections). The middle panel presents the linear regression analysis of the data. The right panel illustrates the schematic diagram of no reflection (1 reflections). (**c**) The left panel shows the voltage fluctuation curves generated when droplets containing solutions of different concentrations pass through the detection zone of the chip without reflection (2 reflections). The middle panel presents the linear regression analysis of the data. The right panel illustrates the schematic diagram of no reflection (2 reflections). (**d**) The left panel shows the voltage fluctuation curves generated when droplets containing solutions of different concentrations pass through the detection zone of the chip without reflection (3 reflections). The middle panel presents the linear regression analysis of the data. The right panel illustrates the schematic diagram of no reflection (3 reflections).

As shown in Figure 4a, when an optical path length of 0.5 mm was used, the incident light simply passed through the droplets and was received by the exit optical fiber without undergoing reflections inside the chip, thus exhibiting a concentration detection limit of 0.1 mM. When the optical path length was 1 mm, the incident light underwent 1 reflection inside the chip. Although the optical path length was increased and the detection performance was better than that with 0 reflections, the concentration detection limit remained 0.1 mM (Figure 4b). For an optical path length of 2 mm, the incident light underwent 2 reflections inside the chip. Compared with 0 and 1 reflections, the increased optical path length improved the chip sensitivity, resulting in a low concentration detection limit of 0.02 mM (Figure 4c). When the optical path length was 3 mm, the incident light underwent 3 reflections inside the chip. Due to the increased non-absorptive loss of light intensity, the intensity of the emitted light signal was significantly reduced, thus only showing a concentration detection limit of 0.2 mM (Figure 4d). Notably, all detection limits here follow the 3σ principle of the blank control group. After the experiments, the absorbance values were calculated using the formula and subjected to fitting analysis. As shown in Figure 3, the R^2^ values of the four groups of measurement data are all >0.98, demonstrating reliable absorbance measurement performance within the laboratory microfluidic setup.

It can be concluded that the microfluidic chip with an optical path length of 2 mm achieves the lowest detection limit (0.02 mM, indicating the highest sensitivity). Therefore, in subsequent detection experiments, the microfluidic chip with a 2 mm optical path length was adopted, see Figure 4.

### 3.3. Microbiological Applications

**Figure 5:**
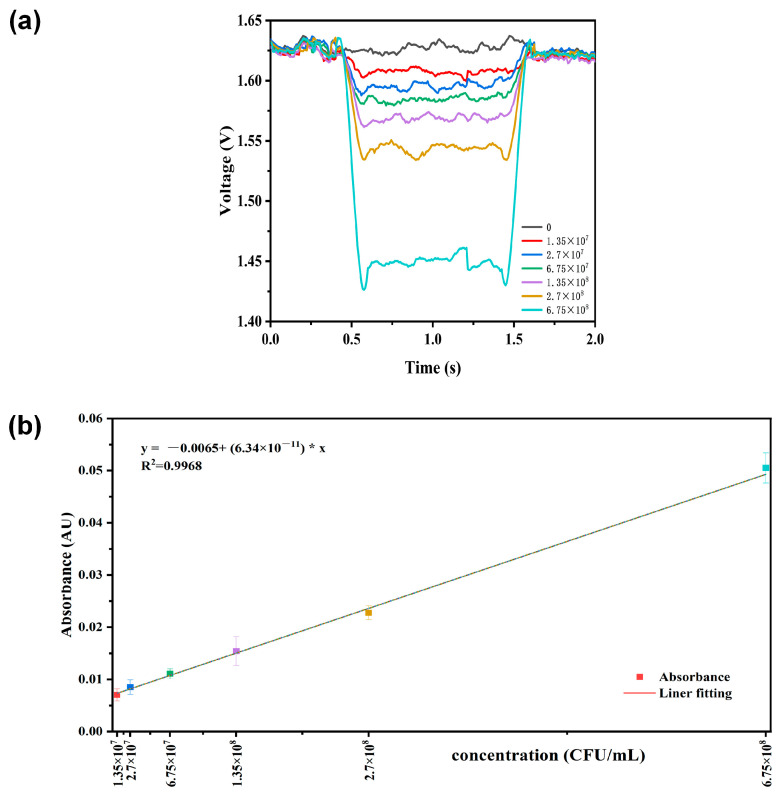

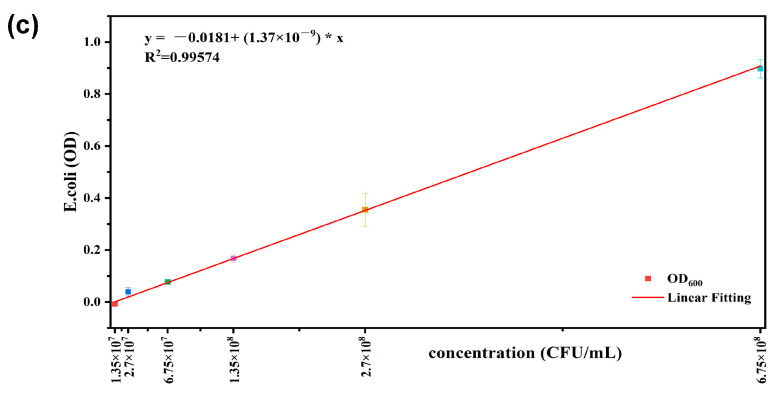
(**a**) Voltage fluctuations generated when droplets containing *E. coli* suspensions of different concentrations pass through the detection zone of the 2-reflection chip. (**b**) Perform linear regression analysis on the test data. (**c**) Linear regression analysis was performed on the OD_600_ data of sample bacterial suspensions measured by Nanodrop.

Based on the characterization and analysis of the microfluidic device, we further tested droplets containing microbial samples. Air was used as the continuous phase, and the prepared *Escherichia coli* (*E. coli*) bacterial suspension served as the dispersed phase. Droplets were generated via the alternating pumping of the peristaltic pump and sequentially passed through the detection zone, with the voltage signal of each droplet measured synchronously to plot the corresponding curve (Figure 5a). According to the 3σ principle, the detection limit of the device was determined to be 1.35 × 10^7^ CFU/mL. The absorbance values of the samples were calculated using the Beer-Lambert law and subjected to regression analysis. As expected, the absorbance signal showed an increasing trend with the rise in bacterial suspension concentration (Figure 5b), with a fitting R^2^ of 0.98.

For comparison, 1 μL of each prepared *Escherichia coli* sample was measured using a Nanodrop spectrophotometer to determine the OD_600_ value. The minimum detectable concentration of the instrument was 2.7 × 10^7^ CFU/mL. When the concentration decreased to 1.35 × 10^7^ CFU/mL, the signal was close to the baseline, indicating limited detection capability. A calibration curve was constructed based on these measurements (Figure 5c). Under the current experimental conditions, the integrated microfluidic chip enabled effective detection of *E. coli* at 1.35 × 10^7^ CFU/mL. Compared with the commercial Nanodrop instrument, the chip with a 2 mm optical path length exhibited approximately a twofold improvement in detection sensitivity for the *E. coli* suspension, demonstrating superior detection performance. Although previously reported microfluidic absorbance detection systems can achieve lower limits of detection, they mostly rely on complex chip structures (Liquid-core waveguide [24]) or sophisticated optical integration schemes(digital microfluidics [27]). In contrast, the proposed chip features a simple fabrication process and low cost, and can achieve the above detection limit without any sample pretreatment or signal amplification. This indicates that the strategy of integrating on-chip micro-mirrors is an efficient and cost-effective approach to enhance the detection sensitivity of microfluidic absorbance measurements.

## 4. Conclusions

Herein, we developed a simple and cost-effective method for measuring droplet absorbance. The key innovation of this work lies in perfusing low-melting-point liquid metal into pre-designed microfluidic channels, where the sidewalls of the microchannels serve as reflectors. The optical path formed by multiple reflections effectively prolongs the absorption path length and enhances the detection sensitivity. Notably, the fabrication process of such on-chip microreflectors is simple and low-cost: the liquid metal costs only 60 RMB, which is far lower than conventional mirror fabrication consumables like gold plating and silver plating, and the entire processing time is merely 0.5 h. The device adopts a microfluidic chip with an optimal optical path length of 2 mm to achieve the best detection performance. Experimental results show that the detection limit for methylene blue solution reaches 20 μM. Meanwhile, this method is applied to measure the concentration of *Escherichia coli* (*E. coli*) suspension. The minimum detectable concentration of the traditional Nanodrop instrument is 2.7 × 10^7^ CFU/mL, whereas this chip achieves a lower detection limit of 1.35 × 10^7^ CFU/mL, representing a doubled detection sensitivity compared with the conventional Nanodrop device. Finally, thanks to its prominent advantages of low cost and simple operation, this device is expected to make breakthroughs in on-site rapid detection scenarios and realize high-sensitivity sample analysis.

## Data Availability

Data is contained within the article or Supplementary Materials.

## Supplementary Materials

The following supporting information can be downloaded at: https://www.mdpi.com/article/10.3390/bios16040202/s1, Table S1: Cost Table of Peristaltic Pumps.
