# Supplementary material for: Droplet-Interlaced Generator with On-Chip Metal–Liquid Micromirrors for Enhanced Microfluidic Absorbance Detection
Source: Biosensors (Basel). 2026 Apr 2;16(4):202. doi: 10.3390/bios16040202 (PMC13114035; doi:10.3390/bios16040202)
Supplement: Supplementary file 1 [file biosensors-16-00202-s001.zip › biosensors-4225266-supplementary.pdf]

Supporting Information for

Droplet-Interlaced Generator with On-Chip Metal–Liquid Micromirrors for Enhanced Microfluidic Absorbance Detection

Haobo Liu[1,2,#], Laidi Jin[3,#], Zehang Gao[2], Chuanjin Cui[1], Yongjie Yu[4], Fei Deng[5], Xiuli Gao[2], Jianlong Zhao[2,6,7], Shengtai Bian[8,9,*], Shilun Feng[1,2,*]

[1] North China University of Science and Technology, No. 21, Bohai Avenue, Tang'shan, 063000, China.
[2] State Key Laboratory of Transducer Technology, Shanghai Institute of Microsystem and Information Technology, Chinese Academy of Sciences, Shanghai 200050, China.
[3] Department of Stomatology, Shanghai Municipal Hospital of Traditional Chinese Medicine, Shanghai 200071, China
[4] School of Materials Engineering, Changzhou Vocational Institute of Industry Technology, Changzhou 213164, China.
[5] Graduate School of Biomedical Engineering, Faculty of Engineering, University of New South Wales, Sydney, NSW, 2052, Australia.
[6] Shanghai Frontier Innovation Research Institute, Shanghai 201108, China.
[7] Xiangfu Laboratory, Jiashan 314100, China.
[8] Agriculture and Forestry Artificial Intelligence Research Institute, Fujian Agriculture and Forestry University, Fuzhou 350002, Fujian, China.
[9] College of mechanical and electrical engineering, Fujian Agriculture and Forestry University, Fuzhou 350002, Fujian, China.

Supplemental Tables:

Table S1 Cost Table of Peristaltic Pumps

Module	Specification	Cost(RMB)
42 Stepper Motor	0.7N	45
Driver	TB6600	30
Peristaltic Pump Head	BT005M-MB-04-P3B3.2G4.0	490
Silicone Tube	1×3mm/2×4mm	6
Coupling	Inner Diameter 5mm	10
D-Shaft	D5mm-D1mm	8
Total		589
